# Hierarchically nanostructured materials for sustainable environmental applications

**DOI:** 10.3389/fchem.2013.00018

**Published:** 2013-11-12

**Authors:** Zheng Ren, Yanbing Guo, Cai-Hong Liu, Pu-Xian Gao

**Affiliations:** ^1^Department of Materials Science and Engineering, Institute of Materials Science, University of ConnecticutStorrs, CT, USA; ^2^Center for Clean Energy Engineering, University of ConnecticutStorrs, CT, USA

**Keywords:** nanomaterials, environmental catalyst, sensors (chemical and bio), hierarchical assembly, sustainability

## Abstract

This review presents a comprehensive overview of the hierarchical nanostructured materials with either geometry or composition complexity in environmental applications. The hierarchical nanostructures offer advantages of high surface area, synergistic interactions, and multiple functionalities toward water remediation, biosensing, environmental gas sensing and monitoring as well as catalytic gas treatment. Recent advances in synthetic strategies for various hierarchical morphologies such as hollow spheres and urchin-shaped architectures have been reviewed. In addition to the chemical synthesis, the physical mechanisms associated with the materials design and device fabrication have been discussed for each specific application. The development and application of hierarchical complex perovskite oxide nanostructures have also been introduced in photocatalytic water remediation, gas sensing, and catalytic converter. Hierarchical nanostructures will open up many possibilities for materials design and device fabrication in environmental chemistry and technology.

## Hierarchical nanostructures: complexity that matters

Since the discovery of carbon nanotube back in 1991, the past two decades have witnessed the rapid development in nanotechnology with numerous nanostructures synthesized and unique properties discovered (Alivisatos, 1996; Hu et al., 1999; Smith and Nie, 2009). The representative nanoscale architectures include nanoparticles, one-dimensional nanowires and two-dimensional nanosheets. With the size comparable to the wavelengths of electrons and photons, the nanostructures usually demonstrate unique physical properties governed by quantum mechanics, which are distinct from those of the bulk materials. For example, the band gap of quantum dots can be tuned by simply size variation (Alivisatos, 1996). One-dimensional metal nanostructures are able to exhibit semiconducting behavior while semiconducting Si nanowire can become insulating (Xia et al., 2003). These newly discovered physical properties associated with nanostructures of controlled geometry has opened up exciting opportunities for new materials design and will potentially revolutionize current device manufacturing techniques. However, it is necessary to achieve the large-scale assembly of these nanoscale units to realize the practical utilization of nanostructures. As the core essence of nanomaterials chemistry, the bottom-up paradigm provides an important guidance that aims at designing and arranging materials at the submicron or nanoscale dimension (Lu, 2007).

In recent years, hierarchical nanostructures composed of either geometric complexity or multiple constituents have drawn great interest (Gao and Wang, 2002; Lao et al., 2002; Whang et al., 2003; Ding et al., 2006; Gao et al., 2012). The hierarchical structures can be categorized into either the structures with nanoscale building blocks extended into more than one dimension or the structures with multiple components. In the first scenario, the accomplished hierarchy of nanoscale building blocks represents the successful materials manipulation and helps reveal important scientific understanding at an unprecedented length scale. The complexity in extended dimensions brings about advantages such as high surface area that is important in water treatment (Savage and Diallo, 2005) and large scale ordered arrangement for electronic device manufacturing (Huang, 2004). For instance, three-dimensional urchin-like ZnS hierarchical spheres demonstrate enhanced photocatalytic activity owing to their enhanced light harvesting efficiency and high surface to volume ratio (Liu et al., 2011a). ZnO nanowires have been successfully integrated into ordered two-dimensional arrays with interesting gas sensing (Wang et al., 2006) and photo-detecting property (Soci et al., 2007). As mentioned above, however, the materials hierarchy can be more than geometric arrangement. Different combination of dissimilar units may lead to multiple functionalities in the hierarchical materials, exhibiting complex property (Sen et al., 2010; Liu et al., 2011b). The development of such hierarchical nanostructures is an important task in advanced nanotechnology to realize better materials performance by rational combination of multiple components.

In the following sections, we will review the design and synthesis of hierarchical nanostructures targeted at sustainable environmental applications. The discussion of hierarchically nanostructured materials will be focused on photocatalytic water treatment, biosensing, environmental gas sensing and monitoring, as well as catalytic polluting gas conversion.

## Hierarchical nanostructures design for water treatment

Water is a very scarce natural resource especially in developing countries due to the lacking of public access and sanitation. In the UN's Millennium Development Goals, it is expected to halve the numbers of people without access to clean drinking water by 2015 (The Millennium Development Goals and Water, 2000). Nanomaterials provide the possibility to facilitate the process by improving the efficiency of typical water treatment which involves oxidation of organic pollutants and removal of toxic ions such as arsenic and chromium (Mills et al., 1993; Ngomsik et al., 2005; Wingenfelder et al., 2005; Hu et al., 2008a; Shannon et al., 2008; Chong et al., 2010; Tong et al., 2012). In this section, we will focus on the latest development of synthetic strategies for various hierarchical nanostructures and discuss their performance in water treatment.

### Photocatalytic degradation of organic contaminants

#### Single-component hierarchical structures: vapor deposition and wet chemical assembly

Semiconductor nanocrystals have been widely used in the photocatalytic redox process because of the electronic configuration of filled valence band (VB) and empty conduction band (CB) (Hoffmann et al., 1995). When exposed to a photon with energy exceeding the band gap, *hv* > *E*_*g*_, it generates an electron-hole pair with one electron in VB pumped into CB leaving a hole behind in VB. The generated holes in VB are of great oxidation capability while the electrons in the CB have high reducing potential. These highly reactive electrons and holes participate in the photocatalytic organic degradation. As typical semiconducting materials, TiO_2_ and ZnO have been demonstrated to be durable candidates in contaminant removal (Turchi and Ollis, 1990; Linsebigler et al., 1995; Chakrabarti and Dutta, 2004) and solar energy conversion (Grätzel, 2005; Saito and Fujihara, 2008; Zhang et al., 2008). Compared with other photocatalysts, both TiO_2_ and ZnO have a number of advantages featuring high catalytic activity, relatively low cost, good chemical stability, and environmental safety. It is thus of scientific and engineering importance to tailor the materials design strategies to promote their photocatalytic performance.

A closer look at the photocatalytic process described above reveals that the catalytic efficiency depends on several key factors. First, the recombination of photogenerated electron-holes pairs deteriorates the charge transport leading to low reaction efficiency at the semiconductor surface. Typically, the recombination takes place within nanoseconds after the generation of electrons and holes by photon illumination and the energy is usually dissipated as heat. The recombination can be facilitated by imperfections. From this perspective, smaller particles with reduced density of grain boundary and better crystallinity are favorable. Second, a high surface to volume ratio is necessary so that the number of catalytic reaction sites is increased. The higher surface area is also able to facilitate the organic molecules adsorption which is favorable for reaction kinetics.

**Figure 1:**
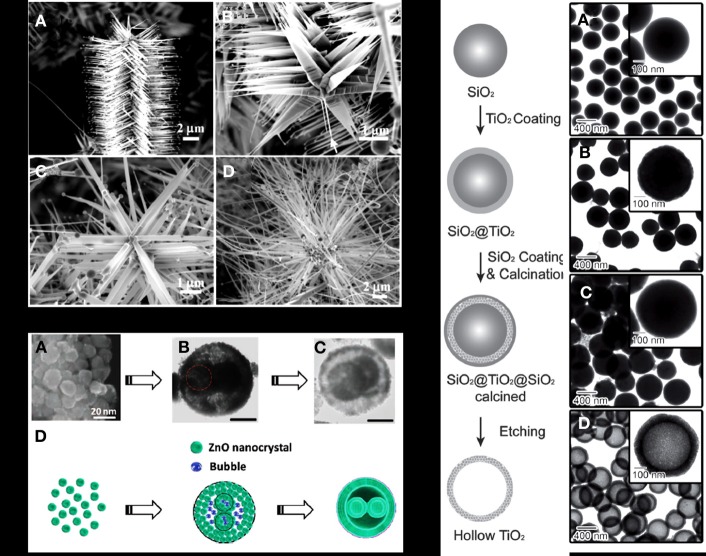
**(A)** ZnO nanopropeller synthesized by vapor deposition; **(B)** Ostwald ripening utilized to prepare ZnO hollow spheres with double-yolk egg architectures; **(C)** Silica sphere templated synthesis for mesoporous anatase TiO_2_ hollow structure. [Reprinted with permission from reference Gao and Wang (2004). Copyright 2004 AIP. Reprinted with permission from reference Wang et al. (2012a). Copyright 2012 Wiley. Reprinted with permission from reference Joo et al. (2012). Copyright 2012 Wiley].

Single-component hierarchical nanostructures with nanoscale building blocks assembled and oriented into more than one dimension enable the efficient spatial utilization of materials property (Gao and Wang, 2005a; Liu et al., 2008a; Tian et al., 2011). For example, propeller-shaped hierarchical structure has been successfully achieved by a solid vapor deposition to extend single crystalline ZnO nanorod into three dimensions (Gao and Wang, 2004). The growth morphology depends on local temperature, surface diffusion, and vapor supply. As illustrated in Figure 1A, a triangle-shaped morphology is achieved in the relatively low temperature region where there is insufficient vapor supply and slower surface diffusion. In regions of higher temperature, uniform and longer blades are formed due to faster vapor diffusion and higher surface mobility. Other hierarchical geometries have been achieved for ZnO nanostructures through vapor phase deposition such as nanosprings (Gao and Wang, 2005b), nanorings (Kong and Wang, 2003; Hughes and Wang, 2005), and nanohelices (Gao et al., 2005). Wet chemical method, which is facile and less energy consuming, has also been utilized in hierarchical ZnO nanostructure preparation. For instance, Wang et al. reported a one-pot synthesis based on Ostwald ripening mechanism as displayed in Figure 1B to prepare ZnO hollow spheres with double-yolk egg architectures (Wang et al., 2012a). Hollow ZnO microspheres can be selectively prepared from the self-assembly of ZnO nanorods and thin nanosheets (Mo et al., 2005).

Free standing TiO_2_ nanoparticles with different architectures have been designed to increase large surface area for photocatalytic degradation. Typically TiO_2_ has three polymorphs including anatase, rutile, and brookite. While rutile TiO_2_ is the thermodynamically stable phase and has relatively small band gap, anatase TiO_2_ exhibits better photocatalytic activity because of its higher reduction potential and lower recombination rate. Hierarchical hollow structure (Fan et al., 2007; Sun et al., 2011a,b) and urchin-shaped architectures (O'Dwyer et al., 2006; Xu et al., 2009; Xiao et al., 2012) represent good designs that tailor the surface to volume ratio. For the preparation of hollow TiO_2_ nanostructures, hard templates including silica and metal oxides are generally used. Joo et al. reported the synthesis of mesoporous anatase TiO_2_ shells with hollow interior by silica templated calcination (Joo et al., 2012). As illustrated in Figure 1C amorphous TiO_2_ deposition on the colloidal silica template was achieved by a sol-gel process. The subsequent calcination at different temperature combined with chemical etching of silica was found to tune the crystalline phase and porosity of the TiO_2_. The as-prepared anatase TiO_2_ mesoporous shells have large surface area as well as good dispersion in water which contribute to rapid degradation of Rhodamine B under UV irradiation. Similar procedure was adopted when silica was replaced by metal oxide. In this scenario, acid etching was applied to remove the metal oxide template. One of the advantages of using metal oxide nanostructures as template is the capability to tune the hierarchical features of the hollow structures. For example, Fe_2_O_3_ nanoparticles of both spindle and cube geometries were used as the sacrificial backbones for the hydrothermal preparation of TiO_2_(Wang et al., 2012b). The Fe_2_O_3_ interior was etched away by the acidic TiF during TiO_2_ crystallization without damaging the architecture geometry. The concurrent crystallization and template etching makes a one-pot synthesis of hollow structures without post treatment. It therefore, provides a facile strategy to tailor the hollow structure geometry by shape variation of the interior templates.

**Figure 2:**
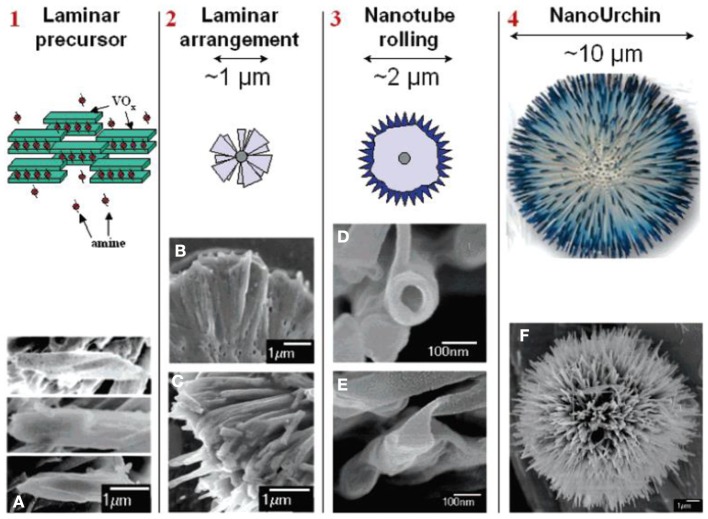
**Schematic illustration of the morphology evolution and the growth mechasnim of vanadium oxide nanourchin. (A–F)**: SEM images of the as-synthesized nanostructures at different growth stages. [Reprinted with permission from reference O'Dwyer et al. (2006). Copyright 2006 American Chemical Society].

Compared with hollow structure that increases the proportion of surface within a confined space, the urchin-like architecture extends active catalytic surface by stretching nanoscale features into more than one dimension (Bakr et al., 2006; O'Dwyer et al., 2006; Xu et al., 2009). The self-assembled urchin-like structure was first reported in the vanadium oxide system, which consisted of spherically arranged nanotube radial arrays (O'Dwyer et al., 2006). In addition to the elongated geometry contributing to high surface area, it is worth noting that the one-dimensional feature of nanowire building blocks will promote efficient electron-hole separation and migration resulting in fast charge transport. Such advantages enabled by three-dimensional arrangements are beneficial for the surface catalytic reaction owing to a relatively low recombination rate. Many urchin structured metal oxides such as SnO_2_ (Jia et al., 2009), In_2_O_3_ (Chen et al., 2009), WO_3_ (Xi et al., 2012) have been obtained by self-assembly in the solution and demonstrate good photocatalytic organic degradation performance. Typically, the formation of urchin-like structures is driven by the minimization of interfacial energy between one-dimensional nanoscale building blocks. In the vanadium oxide system, for example, anisotropic laminar structures were first formed and self-assembled into spherical aggregates in a radially oriented fashion guided by amine molecules (O'Dwyer et al., 2006). Each laminar units constituting the spherical aggregates underwent a rolling procedure in the presence of amine and nanotubes with micrometers in length came into shape with minimized the surface energy. The length of the nanotubes was elongated as the hydrothermal reaction proceeded to achieve the final urchin geometry. Figure 2 illustrated the morphology evolution and growth mechanism. Similarly, in the hydrothermal preparation of In_2_O_3_, (Chen et al., 2009) individual nanowires appeared in the early stage and self-arranged into urchin architecture to reduce the surface energy. Such a two-step growth mechanism, which involves formation of anisotropic building blocks followed by self-assembly driven by surface energy minimization, has been widely used to interpret the growth process of urchin-like structures. However, a different scenario has been observed and different mechanisms have been proposed for the formation of TiO_2_ nano-urchin. Xiang et al. reported the hydrothermal preparation of urchin-shaped TiO_2_ nanoarchitecture where the one-dimensional growth of TiO_2_ was observed in the second stage of reaction (Xiang et al., 2012). Smaller nanoparticles aggregated into large spheres instead of forming one-dimensional building block after nucleation. As reaction continued under hydrothermal temperature of 150°C, these small particles in the solution dissolved and the recrystallization occurred to form rutile one-dimensional branches grown on the surface of microspheres. Similar phenomenon but different mechanism was observed in rutile TiO_2_ urchin formation reported by Park et al. (2012). The submicrospheres aggregated by smaller nuclei appeared in the first stage of reaction at lower temperature. When temperature increased to 90°C, the adjacent small particles in the spherical aggregates underwent a dissolution and recrystallization process to produce single crystalline TiO_2_ as reaction time increased. This process was confirmed by the time-dependent experiments where the size of the spherical aggregates decreased during the growth of rutile TiO_2_ nanorods. Therefore, it can be concluded that the TiO_2_ branches formation is achieved through direct mass transfer from the initial submicrospheres, which is contrary to the secondary growth of TiO_2_ nanorod on the sphere surface described by Xiang et al. previously.

In this section, we focused on the typical vapor deposition method and wet chemical synthetic strategies to achieve high surface area for hierarchical nanostructures. The design principles and the growth mechanism for particles with hollow interior and the urchin-shaped architecture, both of which are typical examples of hierarchical nanostructures, have been introduced. Although the discussion was mainly restricted to the semiconducting ZnO and TiO_2_, which are widely used for photocatalytic degradation of organic molecules in water under ultra-violet light illumination, the synthesis approaches apply to other semiconductor nanomaterials as well.

#### Hierarchical composite nanostructures—I: band gap engineering and plasmonic photocatalysis

Another problem associated with photocatalyst application is the limited light harvesting efficiency. As representative robust semiconducting materials, both ZnO and TiO_2_ have a relatively large band gap (over 3 eV) and therefore, only allow light absorption within the ultra-violet range, which is just a small portion of the solar spectrum. It is therefore, desirable to improve the catalytic activity under solar illumination by either replacing with new materials or adding chemical complexity. Although many semiconducting materials with small bad gap have been demonstrated to facilitate visible light absorption, their UV photo-response is usually sacrificed. Furthermore, they are not as chemically stable and cost-effective as titania. In this section, we will first focus on ZnO-based materials system to introduce band gap engineering by hierarchical composite nanostructures with tailored chemical composition. Later we will discuss the physical mechanism behind plasmonic photocatalyst design which improves the catalytic performance in the visible light region.

**Figure 3:**
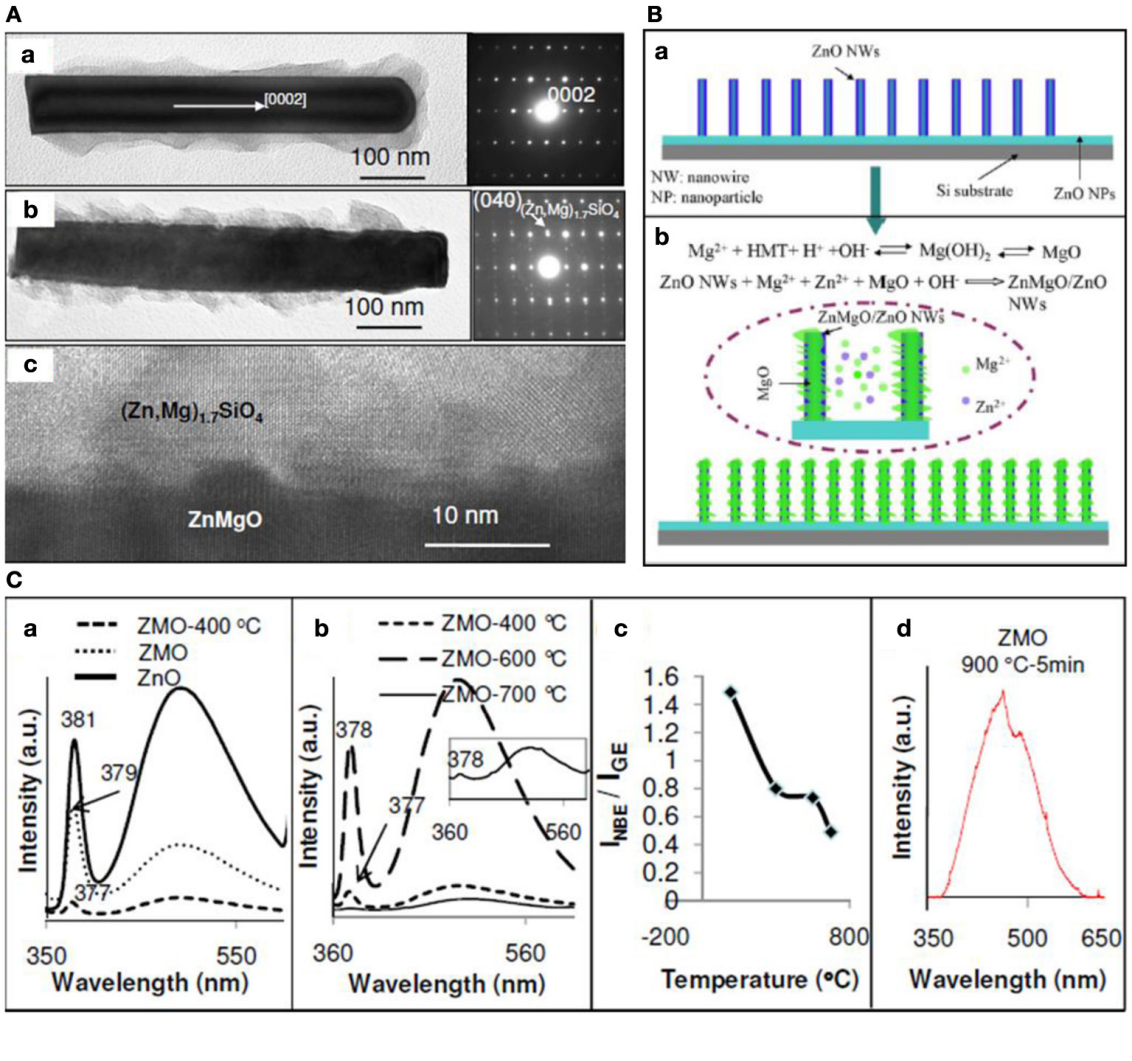
**TEM characterization of ZnMgO nanowire arrays; hydrothermal synthesis and growth mechanism of ZnMgO nanowires; photoluminescence spectra ZnMgO at different temperature. (A)** TEM characterization of ZnMgO nanowires. **(B)** Illustration of the hydrothermal growth mechanism. **(C)** Photoluminescence spectra of ZnMgO treated at different temperatures. [Reprinted with permission from reference (Shimpi et al., 2010). Copyright 2010 AIP. Reprinted with permission from reference Shimpi et al. (2009). Copyright 2012 IOPScience].

A general way for tuning semiconductor's band gap is doping process. Usually the successful doping of ZnO is achieved by a vapor method which requires high temperature. For example, Mn doped ZnO nanowire arrays have been prepared by chemical vapor deposition (Prabhakar et al., 2012). A metal vapor vacuum arc ion doping technique was developed to achieve large scale Ni-doped ZnO nano-arrays (He et al., 2005) and thermal annealing in ammonia atmosphere has been demonstrated to incorporate nitrogen in ZnO nanowires (Yang et al., 2009). Recently, our group has proposed a low-temperature solution strategy to successfully produce ZnMgO nano-arrays (Shimpi et al., 2009). A two-step sequential hydrothermal treatment on ZnO nanowire arrays, as demonstrated in Figure 3, was applied to introduce Mg incorporation. The as-prepared ZnO nanowire arrays were immersed in solutions containing both Zn(NO_3_)_2_ and Mg(NO_3_)_2_ at 155°C and large scale ZnO/MgO dendrite nano-arrays with an interlayer of ZnMgO were developed. The ZnO nanowire arrays on Si substrate served as an effective template that allowed retaining the Mg ion on the surface and facilitated the Mg alloying. Meanwhile, the dendrite amorphous MgO on ZnO nanowire surface promotes the local surface diffusion of Mg ion. The dynamic exchange of surface Zn ion and localized Zn ion in solution also increased the chance of Mg incorporation into ZnO lattice. Post thermal annealing under ambient and vacuum condition was designed to increase the alloying amount (Shimpi et al., 2010). Ambient annealing at 900°C led to the quenching of near-band-edge (NBE) emission and promotes intensity of defect-related band (~505 nm) with red-shift. The quenched NBE emission was ascribed to the formation of epitaxial silicate layer as confirmed by TEM. However, vacuum annealing was found to inhibit the silicate growth and annihilate the defects and therefore, enhanced the NBE emission intensity with quenched defect-related emission in the visible wavelength region.

In addition to traditional doping process, interfacial band structure engineering through heterojunction formation is also studied to enhance the photocatalytic performance under visible light illumination (Fox and Dulay, 1993). Hierarchical mesoporous ZnO loaded with quantum dots was found to exhibit remarkably improved photoactivity by the interfacial charge separation and transfer (Xu et al., 2012). In two-dimensionally assembled ZnO nanowires arrays, hierarchical composite structures are usually prepared by surface deposition of another material. The deposition techniques include pulsed laser deposition (PLD) (Fan et al., 2005), magnetron sputtering (Liao et al., 2011), wet chemical deposition (sol-gel process) (Jian et al., 2009), and atomic layer deposition (Law et al., 2006). We have successfully prepared hierarchical ZnO-perovskite composite nanowire arrays by both sol-gel method and PLD (Jian et al., 2009). The investigation on their photocatalytic performance toward organic molecule degradation will be discussed with more details in the following section that focuses on the perovskite photocatalyst. Hereby we will introduce the preparation of ZnO/CuO core/shell nanowires arrays which exhibit better absorption efficiency in the visible light region (Liao et al., 2011). Cu film was successfully deposited on the surface of ZnO nanowires via magnetron sputtering. Since the conversion from Cu film into CuO coating was achieved by thermal annealing, a systematic study was carried out to understand how the oxygen pressure and flow rate affect the thermal oxidation behavior of Cu nanofilm on the ZnO nanowire arrays. The uniformity of CuO coating depends on the internal oxygen gas diffusion from top to bottom of the nanowire arrays. The relatively high pressure (500 mbar) led to the fast oxidation of Cu film on the top region of the ZnO nanowires and the drastic growth of CuO in this region prevents the further oxygen flowing downward to achieve conformal CuO formation at the bottom of ZnO nanowires. When the oxygen partial pressure was reduced, the CuO coating became more conformal with increased oxygen gas flow rate. Basically the internal diffusion of oxygen molecules in the nanowire arrays is dependent on the difference of pressure at the top and the bottom of the nanowire arrays. The higher flow rate will give rise to large pressure difference that facilitates the oxygen diffusion leading to conformal CuO coating. The better visible light absorption can be attributed to the interfacial band alignment of the ZnO/CuO heterojuction.

**Figure 4:**
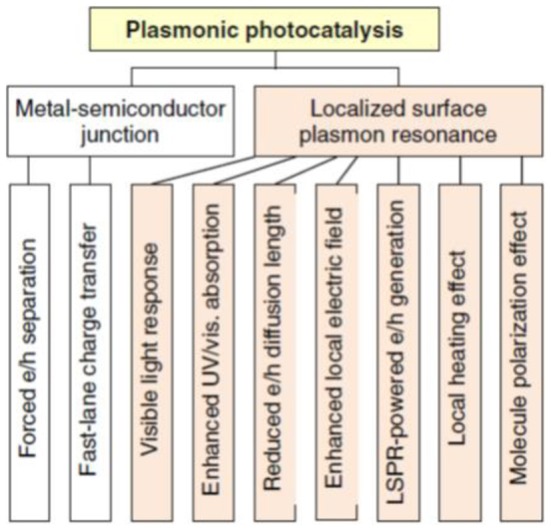
**Summary of advantages of plasmonic photocatalysis.** [Reprinted with permission of reference Zhang et al. (2013). Copyright 2013 IOPScience].

In recent years, plasmonic photocatalysis has drawn increasing attention as a promising technique for highly efficient photocatalytic water treatment (Kowalska et al., 2010; Hou and Cronin, 2013). The advantages of plasmonic photocatalysis have been summarized in Figure 4. Plasmonic photocatalyst is usually prepared by incorporating noble metal nanoparticles such as Au and Ag onto the semiconductor systems (Awazu et al., 2008; Zheng et al., 2011; Wang et al., 2012c). The combination of noble metal and semiconductor leads to enhanced photocatalyst performance under both UV and visible light illuminations. Plasmonic photocatalysis involves two characteristics, Schottky contact and localized surface plasmon resonance (LSPR), both of which directly contribute to the photoactivity (Zhang et al., 2013). Schottky contact forms at the interface of a metal and an n-type semiconductor when the work function of metal (φ_*M*_) is larger than that of the n-type semiconductor (φ_*SC*_). If the semiconductor is p-type the Schottky contact forms when φ_*M*_ < φ_*SC*_. Given the fact that TiO_2_, a widely used n-type semiconductor, has been so far reported to a reliable photocatalyst because of its robustness, low cost, non-toxicity, and excellent photoactivity, we will therefore, restrict our discussion in noble metal-TiO_2_ heterojunctions where Schottky contact occurs. The Schottky contact formed at the interface between noble metal and the semiconductor is able to enhance the migration of photogenerated electrons and holes in opposite directions by the built-in electrical field in the space charge region close to the interface. The electron-hole recombination can be thus, greatly mitigated and the charge transfer would also be facilitated by the noble metal anchored at the surface. On the other hand, the LSPR is defined as the collective oscillation of free electrons on noble metal nanoparticle surface which is in phase with the electrical field of the incident light (Hutter and Fendler, 2004). Since LSPR takes place at the surface of the anchored noble metal and the size of these noble metal nanoparticles is very small, it enables the fast electrons or holes transfer to the surface. In semiconductors of relatively poor charge transfer capability resulting from defects and grain boundaries, the shorter distance to the surface will improve the migration and thus, the photocatalytic reaction efficiency. Besides, LSPR from the existence of noble metal nanoparticles will promote the light absorption efficiency for large band gap TiO_2_. What's more important, the geometry of noble metal nanoparticles including size and shape is going to influence the resonance frequency of LSPR and thus, the photoresponse under different wavelength illumination (Lyon et al., 1999; Jensen et al., 2000). Zheng et al. proposed the design of a core-shell structure with noble metals such as Au, Pt, and Ag coated on TiO_2_ spheres prepared from alcohol-thermal method. The composite Au decorated TiO_2_ particles exhibit the highest yield (63%) and selectivity (91%) for the oxidation of benzene to phenol under visible light illumination (Zheng et al., 2011). In addition to the symmetric core-shell geometry where noble metal nanoparticles are uniformly dispersed on the surface, the anisotropic Janus morphology is more interesting due to the non-symmetric coupling of the metal and dielectric oxide, which can potentially generate extremely strong local electric near-fields (Ghenuche et al., 2008; Lassiter et al., 2008). The Janus Au-TiO_2_ photocatalysts have been demonstrated for efficient visible-light hydrogen generation. The near-fields strongly coupled with the local electronic states of TiO_2_ contribute to enhanced optical absorption and the generation of electron-hole pairs for photocatalysis (Seh et al., 2012).

#### Hierarchical composite nanostructures—II: multiple unit integration and added functionality

During photocatalytic water treatment the homogeneous suspensions of photocatalytic nanoparticles makes it difficult to collect and recycle. In the past decade, several methods have been designed to mitigate this issue. For example, photocatalytic nanoparticles can be loaded onto some chemically-inert substrates such as alumina to facilitate the collection after the water treatment (Pozzo et al., 1997). Besides, the incorporation of magnetic particles was found to help with the separation (Xuan et al., 2008; Ye et al., 2010). However, the light harvesting efficiency could be worsened by introducing inactive substrates because not sufficient light will be shed directly on the catalytic component. With photocatalyst immobilized the interaction between the organic molecules and the catalyst is greatly weakened compared with free standing nanoparticles in the solution. When magnetic nanoparticles are incorporated, the separation process is facilitated. However, the recombination at the magnetic particle–semiconductor interface may degrade the photocatalytic performance (Beydoun et al., 2000). Moreover, the combination with the magnetic particles may decrease the surface area of the photocatalyst which gives rise to less sufficient organic molecules adsorption and lowered catalytic activity. Therefore, the rational design of a robust, high performance and recoverable photocatalyst remains a big challenge.

The possible solution may be the incorporation of highly dispersed magnetic nanoparticles without sacrificing the surface area of photocatalyst by specifically tailored shape and geometry. As we discussed earlier in the single component hierarchical nanostructures, urchin-shaped nanomaterials with high surface area represent a potential candidate (O'Dwyer et al., 2006; Chen et al., 2009; Liu et al., 2011a; Park et al., 2012). The extended one-dimensional nanowires into three-dimensional space could enable more catalytic active sites and higher surface area compared with free standing nanoparticles. The structural advantage of such architecture has been proved in not only water treatment application but lithium ion batteries (Li et al., 2006; Zhou et al., 2011) and solar cells (Jiang et al., 2007; Elias et al., 2010) as well where charge transport is greatly facilitated. Specifically, it is worth noting that the ZnO nanowire assembled hierarchical urchin architecture also enables elongated light path and thus, great performance toward photocatalytic degradation (Wang et al., 2008; Li and Wang, 2009). Recently we have therefore, successfully demonstrated the design of magnetically recyclable ZnO urchin structure (nano koosh ball) with iron oxide submicron particles incorporated as the core component (Ren et al., 2012). A thin layer of amorphous SiO_2_ has been rationally intercalated between the magnetic region and the photocatalytic ZnO nanowires and the combination at the iron oxide-ZnO interface can thus, be greatly suppressed.

**Figure 5:**
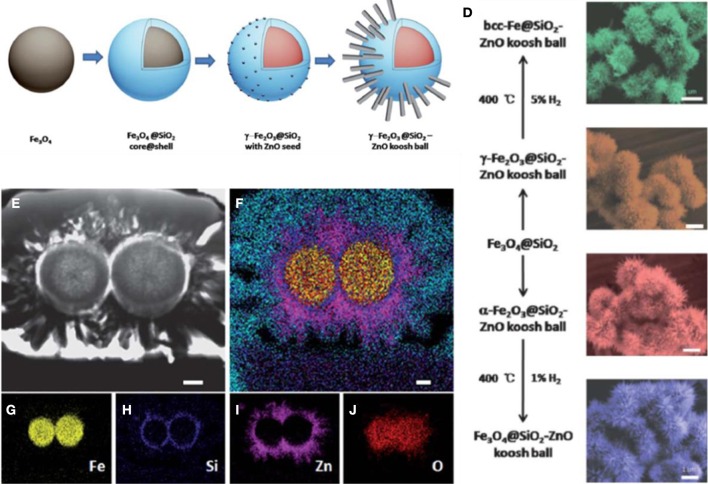
**(A–D)**, Growth process for iron oxide@SiO2-ZnO nano koosh ball; **(E–J)** Elemental mapping of two koosh-ball structures using SEM-FIB dual-beam imaging system; **(K)** Tunable phase transformation of magnetic cores. [Reprinted with permission of reference Ren et al. (2012). Copyright 2012 Royal Society of Chemistry].

As displayed in Figure 5, the synthetic procedure of nano koosh ball involves the hydrothermal preparation of the Fe_3_O_4_ particles in the first step. The classical Stöber sol-gel strategy was applied to deposit amorphous SiO_2_ layer with controlled thickness followed by ZnO seeding process on the surface. ZnO nanowires were then grown out of the seeds on the surface to achieve the final urchin-like morphology. The complex hierarchical nano koosh ball provides the recyclability of ZnO photocatalyst due to the ferromagnetic nature of the Fe_3_O_4_ particles. With the same amount of materials used, the nano koosh balls demonstrated better photocatalytic degradation performance of organic dyes than the pure ZnO powders. The as-prepared hierarchical architecture enables new photocatalyst design with multiple components but added functionality through geometry control by a wet chemical procedure. In addition we further investigated the phase transition of the iron oxide by hydrogen annealing and successfully achieved nano koosh ball with various magnetic cores including bcc-iron, maghemite, magnetite, and hematite. The hydrogen atmosphere annealing was found to not only tune the crystal phase but also adjust the surface defects of ZnO nanowires which led to different photocatalytic properties.

Similar structure design has been proposed by Li et al. in which nest-like layers assembled by titanate nanosheets are wrapped around the magnetic Fe_3_O_4_ core (Li et al., 2011a). The hydrothermally prepared Fe_3_O_4_ particles were coated with SiO_2_ and TiO_2_ in sequence by the sol-gel process. A simple hydrothermal treatment with NaOH upon this double-shell structure etched away the amorphous SiO_2_ interlayer and triggered the simultaneous titanate nanosheets formation from the amorphous TiO_2_ layer dissolution. During this TiO_2_ dissolution process, the energy-favorable heterogeneous nucleation gave rise to the epitaxial titanate nanosheets growth. Because of the two interfaces resulting from the SiO_2_ etching, the nanosheets will grow along the opposite directions from the TiO_2_ dissolution and thus, simultaneously produces two cavities. The advantages of such architecture include high surface area, high photocatalytic activity from the nest like layers and no contact between the magnetic core and photocatalytic shell. Different shell components have been incorporated for the visible illumination photocatalyst with the same adopted architecture. Xi et al. reported the construction of WO_3_ nanowire branches onto the magnetic Fe_3_O_4_ particles surface by the alcoholysis process of tungsten chloride under hydrothermal condition (Xi et al., 2011). The composite nanostructure shows decent photocatalytic degradation of both Rhodamine B and methylene blue under visible light illumination. The recyclability of the photocatalyst has been demonstrated by repeated use for five times without significant loss in catalytic activity.

#### Hierarchical composite nanostructures—III: nanostructured perovskite photocatalysts

Perovskite represents a class of materials with multiple functions including ferroelectricity (Cohen, 1992; Fong et al., 2004), superconductivity (Chu et al., 1987), dielectrics (Homes et al., 2001) and other novel properties targeting different applications in electronics (Mathews et al., 1997), energy conversion and storage (Takahashi and Iwahara, 1971), catalysis (Suntivich et al., 2011), and so on. In traditional ceramic industry, perovskite has been widely studied for dielectrics and piezoelectrics and other coupled properties guided by the structure-property cliché in materials science. Perovskite nanostructures have been demonstrated in the past decade to have great potential in clean energy technology, environmental sustainability, and remediation. Compared with oxide materials, however, perovskite nanostructures have been less investigated mainly due to the limited availability of synthetic strategies. In this section, we will focus on the research efforts in our group regarding the synthesis and property study of perovskite relevant for the photocatalytic water treatment.

**Figure 6:**
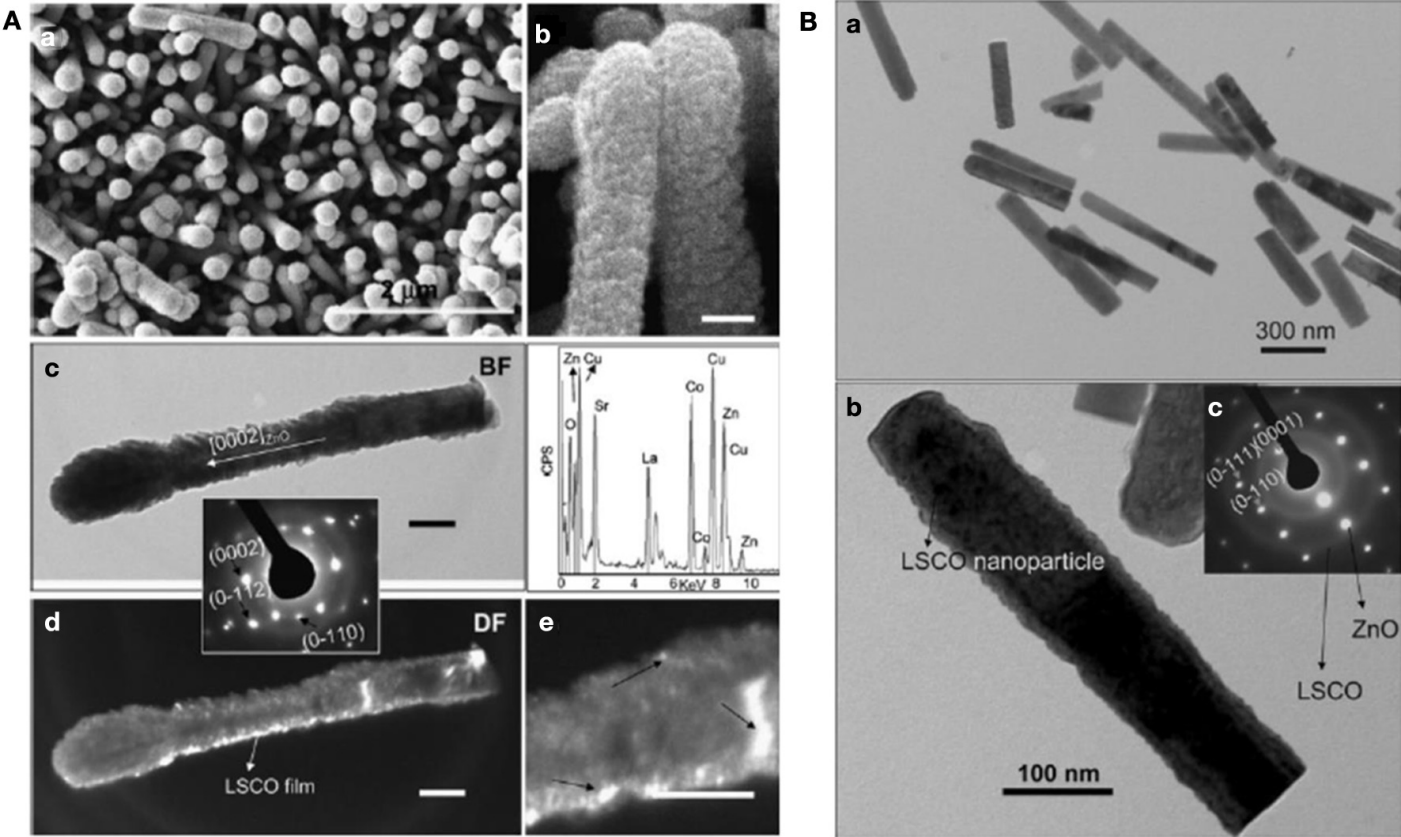
**Electron microscopy characterization of ZnO/LSCO composite nanorod arrays prepared by (A) Pulsed laser deposition; **(B)** sol-gel solution method.** [Reprinted with permission of reference Jian et al. (2009). Copyright 2009 Royal Society of Chemistry].

In our group we successfully prepared hierarchical ZnO/perovskite core-shell nanostructures by a well-developed hydrothermal synthesis followed by either PLD or sol-gel wet chemical process (Jian et al., 2009). The typical electron microscopy characterization of ZnO/LSCO composite nanorods has been displayed in Figure 6. The advantages of PLD over the sol-gel wet chemical method involve fast and conformal deposition, good epitaxy and better crystallinity control. The deposited film uniformity was found to be not only dependent on experimental parameters set for the PLD deposition but also influenced by physical and geometric features of the nanorod diameter and length, assembly density as well as specific surface area of the nanorod arrays. On the other hand, the sol-gel process represents a low temperature facile chemical strategy which requires less energy consumption and easy experimental setup. Relatively uniform deposition can still be achieved onto the nanorod arrays by carefully controlled pH of solution, although a much longer preparation time is needed compared with the fast PLD deposition.

The as-prepared composite hierarchical nanorod arrays were found to show a higher photocatalytic activity toward methylene orange degradation compared with those of pure ZnO nanorod arrays and perovskite thin film samples. Specifically, the enhancement of photocatalytic activity may be ascribed to the increased active sites as a consequence of the high surface area from the mesoporous Sr-doped lanthanum cobalt oxide (LSCO) polycrystalline nanofilm coating. Besides, the ZnO-perovskite interaction at the interface may be relevant as well. From the standpoint of surface area, it is important to control increase the photo-catalytic activity of the ZnO/LSCO composite nanoarchitecture, it is necessary to maximize the surface area of the ZnO nanorod arrays that act as a template in the synthesis by controlling the array density, geometric size and shape, spacing between individual nanorods. Secondly, a comprehensive study upon the LSCO thin film deposition is necessary for the optimum composition of LSCO, porosity control and the better interface structure design.

The immediate question we need to address is the roles both ZnO and the perovskite coating play during the photocatalytic process. Decoupling the functionality from dissimilar components is critical to understand whether synergic interaction exists at the ZnO-perovskite interface. Specifically in the scenario of ZnO-LSCO composite nanowires array, a strict comparison regarding the photocatalytic performance and electronic structure should be directly performed in three hierarchical nanostructure systems: pure ZnO nanowire array, ZnO/LSCO composite nanowire array and hollow pure LSCO nanotube array with the same geometric size control. The difficulty is therefore, the preparation of LSCO nanotube array. Although there are a few literatures reporting the synthesis of perovskite nanostructures, only free standing nanoparticles or nanowires have been discussed and no wet chemical strategy has been demonstrated to achieve two-dimensional arrangements of complex oxides into ordered arrays. It is worth noting, however, that the hollow structures were first fabricated through template synthesis followed by the template removal. The solution in this case therefore, lies in the removal of ZnO after the deposition of perovskite. We recently proposed temperature-programmed hydrogen reducing (TPR) method to successfully remove the ZnO interior in the hierarchical composite nanowires leaving perovskite nanotube arrays on the substrate (Zhang et al., 2012a). The reducing temperature of ZnO is determined to be 550°C which is far lower than that of the perovskite. The beauty of this process is that hydrogen reducing of ZnO produces Zn metal and water, both of which are in the vapor phase at the temperature higher than 550°C. The products can thus, be easily eliminated by carrier gas in the TPR process. The major advantage of this procedure is the *in-situ* hydrogen reducing only removes the ZnO interior without damaging the chemical composition and the geometry of the perovskite, which offers a reasonable and strict comparison to understand the interface between ZnO support and the perovskite coating. XPS investigation and related band structure construction confirm the strong interaction at the interface which contributes to the enhanced photocatalytic performance compared with pure ZnO and LSCO hierarchical structures. This reducing method may provide possibilities for other hierarchical hollow structure design if the direct chemical synthesis without template is challenging.

### Toxic ion removal and trace detection biosensors

In addition to organic contaminants, the inorganic toxic ions impose another threat to the drinking water quality as well. Generally, toxic ions includes heavy metal ions such as Pb^2+^, Cd^2+^, Cu^2+^, Ni^2+^, Hg^2+^, and As^3+^. These toxic ions can be removed by some well-developed techniques including chemical precipitation, reverse osmosis, membrane filtration and adsorption technology and so on (Fu and Wang, 2011). Adsorption technology has various merits including cost-effectiveness, relatively high efficiency, and convenient utilization, which make it one of the most promising approaches for ions removal from contaminated water. The efficient adsorption materials are usually materials of porous structure associated with a large surface area. For example, charcoal has been used in the water filtration for hundreds of years. In modern days, activated carbons with a broad range of pore sizes resulting from intrinsic cracks are most commonly utilized. Many literatures regarding the traditional toxic ion absorption by carbon-based materials have been published (Kadirvelu et al., 2001; Meena et al., 2005). We will therefore, exclude carbon-based materials in the following discussion but only concentrate on other inorganic hierarchical nanostructures mainly metal oxide designed for better toxic ions removal.

The nanostructured materials designed for metal ions trapping and absorbing has received great research interest and many typical nanostructure systems have been demonstrated since the end of last century. Basically, the advantages “nano” could provide include high surface to bulk ratio associated with high porosity, large metal ion absorbing capacity, and good regeneration ability. In this section, we will review the latest progress in nanomaterials development for ion trapping and adsorption and elaborate the rational hierarchy design.

The selection and design for absorbent materials are required to meet several criteria as follows. First, the absorbent itself should be non-toxic in nature. Second, the absorbent should have selectivity and sensitivity as high as possible toward specific type of ion in addition to the large adsorption capability. Third, the adsorbed ions must be easy to collect off the surface of absorbent materials so that the absorbents could be used for repeated times.

Many metal oxides are low cost and non-toxic materials and the hierarchical nature makes them promising for ion removal in the waste water. Typical examples include magnesium oxide (MgO) (Schiller and Khalafalla, 1984), aluminum oxide (Al_2_O_3_) (Zhang et al., 2012b), iron oxide (Fe_2_O_3_) (Gadde and Laitinen, 1974; Phuengprasop et al., 2011), titanium oxide (TiO_2_) (Chen et al., 2012), and so on. Much research progress has been made in the synthesis of these metal oxides with controlled nanoscale morphology (Hua et al., 2012). Yu et al. reported the synthesis of hierarchical micro/nanostructured MgO which exhibits excellent As(III) and As(V) removal capability (Yu et al., 2011). Different amount of potassium carbonate was found to tune the structure from flower-like shape to nest-like geometry. It is generally alleged that the removal of As(V), which is less toxic compared with As(III), is easy to achieve. However, the hierarchical MgO microstructure demonstrated large As(III) trapping and adsorption. By detailed XPS analysis, the whole adsorption process involved formation of Mg(OH)_2_ by partial reaction of MgO with the aqueous environment, which provided excessive adsorption sites. The adsorption capacities of MgO with both shapes are proved to be higher than similar hierarchical structures of other materials.

Hierarchical iron oxide nanostructures have been extensively studied among all the metal oxide nanostructures for the heavy metal ion removal (Zhong et al., 2006; Li et al., 2011b; Cao et al., 2012). In addition to the large adsorption capacity, high surface area and low cost, an important advantage over other metal oxides is the magnetic property which makes it easily recoverable from water after toxic ion removal. Magnetite (Fe_3_O_4_) and maghemite (γ-Fe_2_O_3_), which are ferromagnetic and ferrimagnetic in nature, have been proved to act as efficient ion absorbents. The relatively strong magnetism makes them promising as magnetic absorbents which can be recycled by small magnetic gradient. Various hierarchical structures including hollow spheres and nanoflowers have been achieved by facile wet chemical approach. The removal efficiency was found to be influenced by particle size. For arsenic ion removal, for example, the efficiency increased by orders of magnitude with the reduced particle diameter. However, the nanoparticles are going to aggregate in water when the size is small and surface modification is thus, important. The humic acid coated Fe_3_O_4_ nanoparticles reported by Liu et al. were found to be stable in tap water, natural waters, and acidic/basic solutions ranging from 0.1 M HCl to 2 M NaOH with low leaching of Fe (<3.7%) and HA (<5.3%) (Liu et al., 2008b). Zhong et al. synthesized self-assembled flower-like iron oxide nanostructures (Zhong et al., 2006). Different calcination conditions were applied to realize the phase transformation that leads to the formation of α-Fe_2_O_3_, γ-Fe_2_O_3_, and Fe_3_O_4_, all of which exhibit good adsorption capability in Ar(V) and Cr(VI) removal. Specifically, the as-prepared iron oxides have larger surface area compared with their bulk counterparts. The removal capacity for Ar(V) (4.65–5.3 mg/g) is slightly better than that of commercial TiO_2_ powders (4.11 mg/g). In the Cr(VI) adsorption, however, the removal capacity of three types of iron oxides is nearly doubled compared with TiO_2_.

In addition to heavy metal ions, the majority of water and soil contamination is of organic compounds, which impose severe threat to environmental and human health. It is therefore, of high necessity to develop environmental sensors of high sensitivity and selectivity that detect trace organic compound(s) rapidly and accurately (Rogers, 2006; Badihi-Mossberg et al., 2007; Zhang and Fang, 2010). Traditional analytical chemistry strategy is usually time consuming and less cost-effective in spite of good analytical accuracy. Biosensing technique enabled by nanomaterials represents a low cost, simple, and fast analytical method that complements the current traditional analytical instrumentations for trace organic compound detection and monitoring (Wang, 2005; Zhang and Fang, 2010; Aragay et al., 2012).

The biosensors can be categorized into several types based on the working mechanisms and the transducing components. Typical biosensors include enzyme and antibody based biosensors. Enzymatic biosensors have several advantages such as stable source of materials, tunable catalytic property, and surface selectivity enabled by gene engineering and modulation of enzyme activity (Rogers, 2006). On the other hand, antibody-based biosensors are of higher precision and selectivity toward certain pollutants since antibodies are able to specifically bind to individual chemical compound or group. Besteman et al. demonstrated the enzyme coated carbon nanotube could act as a single molecule biosensor (Besteman et al., 2003). The attachment of the redox enzyme glucose oxidase (GOx) to the nanotube sidewall was found to induce a clear change of the conductance. With increased complexity in geometry, hierarchical nanostructures have been proved lately to work as potential candidates for biosensor devices. For example, Wei et al. presented an enzymatic glucose biosensor using ZnO nanorod arrays (Wei et al., 2006). The negatively charged GOx was successfully immobilized on positively charged ZnO nanorods through electrostatic interaction. The response time for such prototype biosensors is less than 5 s and the glucose concentration detection limit reaches as low as 0.1 mM. In addition to semiconductor nanowires, hierarchical metallic nanowire array has also been demonstrated to work for glucose detection. Delvaux et al. prepared gold nanotube arrays and GOx was immobilized to the modified gold surface by cross-linking or covalent attachment (Delvaux and Demoustier-Champagne, 2003). Recently, much research effort has been focused on one-pot self-assembly of bionanocomposite which can be directly applied to biosensing application. Zeng et al. reported the self assembled graphene–enzyme hierarchical nanostructures in aqueous solution for electrochemical biosensing (Zeng et al., 2010). The bionanocomposite consists of sodium dodecyl benzene sulphonate (SDBS) functionalized graphene sheets and horseradish peroxidase, which exhibits high electrocatalytic activity toward H_2_O_2_ detection with high sensitivity, low detection limit and fast response. Shao et al. demonstrated single wall carbon nanotube functionalized with IGF1R-specific and Her2-specific antibodies for breast cancer cell detection (Shao et al., 2008).

Different nanostructured biosensors have been proposed based on different working mechanism and physical principles. For example, surface plasmon resonance, an important phenomenon taking place on metal nanoparticle surface as described in previous section of plasmonic photocatlaysis, has also found great potential in biosensor application for trace amount of molecules detection (Shankaran et al., 2007). Other physical principles for biosensors include chemically sensitive field-effect transistors (Janata, 1994) and microcantilevers (Hansen and Thundat, 2005). An apt example is the silicon nanowire developed by Lieber group (Patolsky et al., 2006). The characteristic nanoscale feature of Si nanowire enables higher sensitivity than traditional planar FET sensors because the nanoscale dimension is comparable to the size of biomolecules. The intrinsic oxide coating surface facilitates the linking of receptors on the surface. When such nanowire based sensors are put into specific solutions with certain macromolecules, the binding will lead to changes of surface charges on the nanowire, therefore, the conductance variation easily detected through direct electrical measurement. Avarez et al. reported a nanomechanical biosensor to detect pesticide DDT by measuring the tiny bending of a microcantilever produced by differential surface stress (Alvarez et al., 2003).

## Hierarchical nanostructures for environmental gas sensing, monitoring, and catalytic treatment

Gaseous chemical detection and treatment represents another important task in environmental chemistry (Yamazoe, 2005). The development of gas sensor and catalytic converter for environmental monitoring and remediation plays a critical role in public health and safety (Sadik et al., 2004), household security (Kawamura et al., 2005), automobile design (Ruiz et al., 2003), defense technology (Akyildiz and Kasimoglu, 2004), and aerospace engineering (Bévenot et al., 2000). Despite the fact that the materials selection and the device configuration might vary from case to case toward different applications, the core requirements for a gas sensor or catalytic converter remain the same. For a gas sensor, the device should have both high sensitivity and good selectivity in order to detect trace amount of an explosive, hazardous, or other critical gaseous chemicals against a complex dynamic background. The high sensitivity will guarantee a quick response to trigger the alert while the great selectivity will promote the precision of that response. Besides, the gas sensor should be reliable for long term utilization with maintained sensitivity and selectivity. It therefore, requires the sensing materials to have mechanical robustness and thermal stability. For a catalytic gas converter which is mounted on automobile especially, the key issue is to reduce or eliminate the usage of noble metals without sacrificing the conversion efficiency. On the other hand, it is expected to have the lower work temperature of catalytic converter for less energy consumption.

In the first two parts of this section, we will focus on environmental gas sensing and monitoring by discussing fundamentals regarding basic working mechanisms and device fabrication. The latest progress in gas sensor design by hierarchical nanostructured materials will be reviewed. In the second section, we will concentrate on structured catalyst with nanoscale building blocks toward toxic gas treatment.

### Gas sensors—I: nanostructures and sensing mechanisms

The conventional gas sensor fabrication has witnessed a revolutionary transition from powder based thick film preparation to later thin film technology. Compared with powder thick film technology, thin films prepared by physical or chemical vapor deposition enable more controls over the materials microstructures such as grain size and boundary. Generally, gas sensors are composed of pristine or doped metal oxide with semiconducting, photoconducting, piezoelectric, pyroelectric, or luminescent properties, which can realize transduction upon external stimuli in different ways (Azad et al., 1992). In this review we will only discuss conductometric semiconducting gas sensors since they are cost-effective, flexible in manufacturing and most widely used with a large number of detectable gases. The performance of thin film sensors, however, is reversible and reproducible only when relatively high temperature is maintained. This is because the variation in environmental temperature usually leads to grain coalescence and grain boundary modification which largely affect the electrical property of the semiconducting thin films. The development in nanofabrication has allowed large scale integration of nanoscale building blocks in a controlled fashion that offers possibility to manipulate the microscopic geometry and physical properties. Different semiconducting nanowire arrays of single crystalline nature have been prepared by either chemical vapor deposition or low temperature solution approach. The single crystallinity of semiconductor nanowires is able to improve the thermal stability without grain coalescence and grain boundary alteration. Moreover, the adjustable spacing between each individual nanowire allows for porosity manipulation in a controlled way, which is challenging in thin film technology, to achieve better gas sensing performance.

Other advantageous features of hierarchical nanostructures include the high surface-to-volume ratio, which indicates a large fraction of atoms on the surface making surface reaction more kinetically favorable (Sun et al., 2012). Secondly, the nanoscale dimensions of the building blocks are very close or at least comparable to the Debye length, which measures the field penetration depth in the materials. For example, in most semiconducting oxides the Debye length is usually around several nanometers. When the nanoparticle size or the nanowire diameter approaches this dimension, space charge region will constitute majority of the particle interior or nanowire cross section (Wang et al., 2010). With most of the electrons trapped in the surface states, the electronic property of both nanoparticles and nanowires is thus, going to be greatly influenced by the surface process. Such phenomenon is critical to achieve higher sensitivity in the gas sensor design. In the conductometric semiconducting gas sensors, the sensitivity is defined as:

$$\begin{array}{l} S=\frac{R_{g}}{R_{a}}\text{ for oxidative gas sensing}\\ S=\frac{R_{a}}{R_{g}}\text{ for reducing gas sensing}, \end{array}$$

*R*_*g*_ is the resistance of gas sensing materials at the target gaseous atmosphere, which is either reducing or oxidative, while *R*_*a*_ stands for the resistance of gas sensing materials in the ambient atmosphere, which is air condition in most situations.

The gas sensing mechanism in conductometric semiconducting metal oxide sensors is illustrated by using n-type semiconductor as an example. Generally the trapping of electrons in the surface state upon gas molecules adsorption and the consequent electronic band changes contribute to the conductivity variation. When the n-type semiconductor is exposed to the air, certain oxygen gas molecules are adsorbed on the surface with various extent of coverage depending on the surface condition. These adsorbed oxygen molecules are going to extract electrons from the metal oxide surface and lead to a band structure bending upward at the surface. In semiconductor physics, this created electron-depleted area from band bending is called the space charge layer. The conductivity of the n-type semiconductor with surface trapped electrons is thus, decreased compared with the semiconductor of a clean surface. However, when a reducing type of gas or another oxidative gas is introduced to the surface, the surface reaction with the reducing gas or the competitive adsorption from the other oxidative gas molecules is able to change the original oxygen molecules coverage, which results in the band structure completely restored or less bended. Therefore, the conductivity of the gas sensing semiconductor is recovered to different degree and triggers the resistance change.

### Gas sensors—II: materials design and applications

As discussed above, much research effort has been devoted to the metal oxide based semiconductors for the gas sensing applications. Wang et al. categorize these metal oxides for gas sensing application into two groups which are transition metal oxide and non-transition metal oxides (Wang et al., 2010). The first type of non-transition metal oxides is pre-transition metal oxides such as MgO and Al_2_O_3_ which demonstrates little electronic conductivity with a large band gap and these materials are therefore, rarely used in gas sensing. The other type of non-transition metal oxides is post-transition metal oxides in periodic table. Typical examples involve Ga_2_O_3_ and SnO_2_. The transition metal oxides are more sensitive to the environment because of their multiple valence states. With the d orbitals empty or fully occupied, metal oxides such as TiO_2_, V_2_O_5_, ZnO, WO_3_, and SnO_2_ represent widely used gas sensing materials for reliable performance (Wan et al., 2004; Ponzoni et al., 2006; Zuruzi et al., 2006; Young-Jin et al., 2008). Based on the gas sensing mechanism discussed previously, one of the key parameters for designing high performance gas sensing materials is large surface area and porosity. Hierarchical nanostructures such as hollow architectures and flower shaped structures will satisfy the requirements (Lee, 2009). Typical synthetic strategies including both templated growth and self-assembly to prepare architectures with hollow and flower-like geometry have been introduced in the photocatalytic water treatment section. We will therefore, review the gas sensing performance to illustrate the improvement enabled by these typical geometries. In addition, gas sensors targeted at different toxic gas detections will be covered.

For organic gas sensing, Huang et al. developed hierarchical SnO_2_ nanostructure with porous flower-like shape by a facile hydrothermal method followed with thermal annealing (Huang et al., 2010). The hierarchical SnO_2_ nanoflowers exhibit good response and reversibility toward several organic gases including ethanol, n-butanol, methanol, 2-propanol and acetone at different working temperatures. The sensitivity and response time is better compared with solid thin film, porous thin film and the nanoparticles. Zhang et al. reported the one-pot synthesis to prepare the Cu_2_O hollow spheres with multiple shells (Zhang et al., 2007). The as-prepared hierarchical Cu_2_O had the sensitivity of 1.9 toward 10 ppm ethanol with no significant decay detected after more than 100 cycling tests. The high sensitivity can be ascribed to the improved surface accessibility enabled by multiple layers. These works well illustrates the advantages hierarchical nanostructures have brought in high performance gas sensor design.

Gas sensor development for inorganic toxic gas detection is of crucial importance to public health and safety. Toxic gaseous chemicals usually include hydrogen sulfide, ammonia, carbon monoxide, nitric oxide and flammable hydrogen etc. The detection of these toxic gases should have sufficiently high sensitivity because trace amount of some gases will impose severe health threat or even cause death. Ponzoni et al. fabricated the WO_3_ nanowire networks by thermal evaporation of tungsten powders at 1400–1450°C in the presence of oxygen (Ponzoni et al., 2006). The three-dimensional hierarchical WO_3_ nanowire networks display ultrahigh sensitivity toward NO_2_ detection with concentration as low as 50 ppb. The decent selectivity at different working temperatures is observed for various gases including ammonia, carbon monoxide and hydrogen sulfide. Chen et al. prepared porous ZnO nanoflakes by a microwave hydrothermal method and demonstrate the hierarchical structure exhibits better NO_2_ sensing performance compared with ZnO nanoparticles (Chen et al., 2011). The nanoflakes show high selectivity toward NO_2_ detection with concentration of 0.5 ppm in the atmosphere accompanied by 85 ppm other gases including ethanol, carbon monoxide, propylene, methane, diethyl ether, formaldehyde, acetone, cyclohexane, and dimethylbenzene, some of which are from automobile emission as air pollutants. For trace detection of toxic H_2_S, Kaur et al. used a physical evaporation method to prepare the hierarchical In_2_O_3_ nanostructures with whisker and bipyramidal shapes (Kaur et al., 2008). The single crystal whiskers were found to detect very low concentrations (200 ppb) of hydrogen sulfide. Larger response of single crystal whiskers may result from the presence of defects formed during their growth.

**Figure 7:**
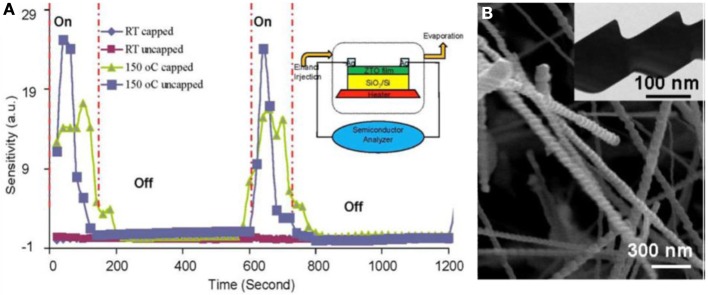
**Ethanol sensor configuration and property based on Ag_2_O/Zn_2_SnO4 hierarchical composite nanowires prepared by vapor solid solid method. (A)** Ethanol sensor configuration and gas sensing performance of Ag_2_O/Zn_2_SnO_4_ hybrid periodic nanowires. **(B)** SEM and TEM(inset) characterization of Ag_2_O/Zn_2_SnO_4_ hybrid periodic nanowires. [Reprinted with permission of reference Cai et al. (2010). Copyright 2010 Royal Society of Chemistry].

A common strategy to enhance the sensitivity of the gas sensors is to incorporate noble metal nanoparticles such as Pt and Au to the hierarchical nanostructures. In carbon monoxide sensing, for example, Au nanoparticles are deposited onto the ZnO nanowire surface and the sensitivity with 50 ppm inlet CO gas can be enhanced from 4.2 to 46.5% at 350°C (Shoou-Jinn et al., 2008). The role of Au nanoparticles played during the gas sensing process is to improve the interaction of adsorbed oxygen with the reducing CO gas. Basically, the noble metal enables preferred adsorption and activation sites for the target gas; the activated atoms can thus, be spilled over onto the semiconductor and react with the adsorbed oxygen. More details on the function of noble metal in gas sensors can be found in a nice review article by Kohl (1990). However, there are other methods to improve the sensitivity without catalyst decoration on the surface. For instance, oxygen plasma treatment can be applied to lower the carrier concentration in the ZnO nanowires and result in a larger population of the O^2−^ ions on the nanowire surface (Law and Thong, 2008). With the reduced conductance, the oxygen plasma treated ZnO nanowires demonstrate four times higher sensitivity toward ammonia detection compared with the ZnO nanowires before treatment. The increased availability of surface O^2−^ ions will contribute to more effective NH_3_ to O^2−^ ion interaction and enhance the sensitivity during gas sensing. Hierarchical composite nanostructure with multiple metal oxides configuration provides another possibility to improve sensitivity. Kim et al. prepared the CuO/ZnO by deposition of CuO nanostructures on the hydrothermally grown ZnO nanorods using a photochemical method (Kim et al., 2012). Compared with bare ZnO nanorods, the composite nanostructure display much improved gas sensitivity. The H_2_S gas response of CuO/ZnO nanorod sensors are found to increase exponentially with temperature. The chemical conversion of CuO to Cu_2_S during H_2_S sensing is responsible for the enhanced gas sensing. Recently we prepared large scale Ag_2_O/Zn_2_SnO_4_ hybrid periodic nanowires at 650°C by using a unique one-step silver oxide catalyzed vapor-solid-solid (VSS) growth process and the ethanol sensing property was investigated (Cai et al., 2010). Figure 7 presents the sensor configuration and ethanol sensing performance of the hybrid periodic nanowires. These Ag_2_O/Zn_2_SnO_4_ hybrid nanowires were found to respond to ethanol at 150°C upon 150 ppm ethanol pulses by one order of magnitude reduced conductivity. This abnormal behavior is ascribed to ethanol pulses associated with Ag_2_O nanoparticles on nanowire surfaces that leads to a catalytic activation of ambient oxygen detection. The 150 ppm ethanol pulse frees a large number of surface donors due to the bounded ambient oxygen molecules at 150°C, which therefore, activated the metal oxide surface sensing activity to the ambient oxygen. With the removal of Ag_2_O nanoparticles by argon plasma treatment, the detection of ethanol molecules instead of ambient oxygen is enabled. This reversible catalytic ambient ethanol/oxygen detection mechanism can be potentially used for gas sensor design targeted at multiple transient gas detection.

**Figure 8:**
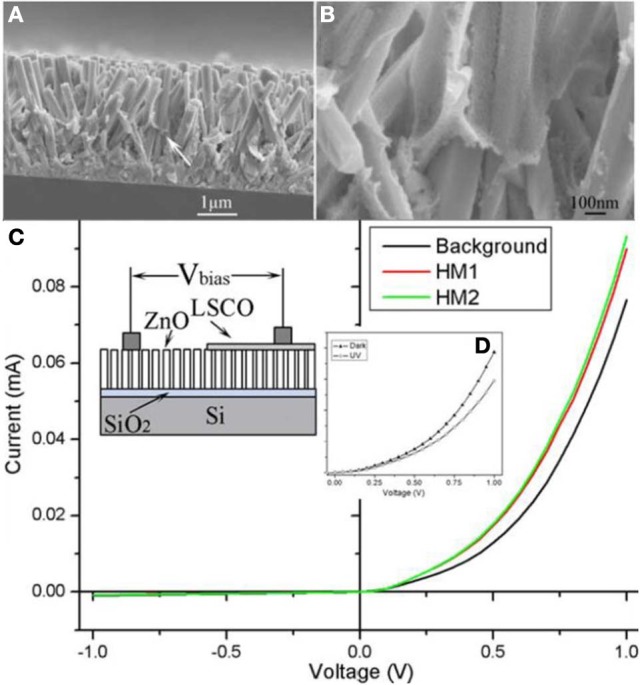
**Humidity sensor configuration and property based on ZnO/LSCO hierarchical composite nanowires array prepared by a combination of hydrothermal and colloidal deposition method. (A,B)** Cross section view of ZnO/LSCO core-shell nanowires array by SEM. **(C,D)** Magnified SEM images of ZnO/LSCO core-shell nanowires array. [Reprinted with permission of reference Gao et al. (2010). Copyright 2010 IOPScience].

Hierarchical composite nanostructures can also be utilized in humidity monitoring and detection. Similar to the ZnO/LSCO photocatalyst preparation, we have grown ZnO/LSCO core-shell nanorod arrays using facile hydrothermal growth followed by the sol-gel deposition (Gao et al., 2010). The uniform coating of perovskite is achieved by carefully controlled solution pH. A good rectifying characteristic have been observed in the ZnO/LSCO heterojunction arrays, which is similar to the characteristic of p-n junctions in semiconductors. As demonstrated in Figure 8, negative photoconductivity response is detected upon UV illumination on the diode arrays resulting from desorption of surface absorbed water moisture. Besides, the forward current of the LSCO/ZnO nanofilm–nanorod diode increases significantly with increasing relative humidity. With increasing humidity, more and more water molecules will adsorb onto the oxide surface through hydrogen bonding. The conductance thus, can be increased according to Grotthuss's chain reaction (Wenmin and Jörg-Uwe, 1997).

In this section, we reviewed the gas sensor development based on hierarchical nanostructures. The gas sensing mechanisms and relevant issues associated with high sensitivity and selectivity have been discussed. Either semiconducting metal oxide with various geometry or hierarchical composite structures have been introduced for various toxic gas detection. The gas sensitivity and selectivity can be improved by noble metal decoration as well as multiple components integration. However, there are still challenges in gas sensor development especially for gas sensing and monitoring in harsh environment. These conditions include high temperature sensing in power plant and combustion engine, gas regulation in automobile emissions as well as gas monitoring in radioactive environment such as nuclear reaction. Recently, we fabricated high temperature O_2_ and CO sensors operating at 800 and 1000°C using CeO_2_ nanofibers (Liu et al., 2012a). The real time *in situ* gas detection is realized with sensitive, reversible, and reproducible response. Similar high temperature oxygen sensor was also prepared using Sr-doped lanthanum manganese oxide (LSMO) nanofibers by electrospinning technique (Liu et al., 2012b).

### Gas treatment: nano-array based catalytic converters

The air pollution has become a worldwide crisis resulting from burning of fossil fuels. A major portion of the air pollutants comes from the automobile exhaust and power plant. The waste gas usually constitutes nitrogen oxide and sulfur dioxide, which either triggers photochemical pollution (Atkinson et al., 1982) or contributes to acidic rain (Mohnen, 1988), as well as hydrocarbon and carbon dioxide, which lead to green-house effect (Rodhe, 1990). Specific catalyst is needed to deal with each pollution gas component. In this section we will discuss the catalysts which deal with the major components contributing to air pollution such as carbon monoxide, hydrocarbons, and nitrogen oxide. The catalytic mechanisms, catalytic techniques as well as the latest progress in preparation of nanocatalyst toward different gas treatment will be reviewed in the first part. Within all the discussion we will introduce the materials selections and discuss how nanoscale geometry matters. Later we will concentrate on catalytic reactor design which rationally integrates the free standing nanocatalysts ranging from two-dimensional planar arrays to a much complex three-dimensional hierarchical fashion.

The best way to treat carbon monoxide and hydrocarbons is the catalytic oxidation. Catalytic CO oxidation has been extensively studied either from catalyst design or reaction mechanism interpretation (Haruta et al., 1993; Lopez and NøRskov, 2002; Kung et al., 2003; Lopez et al., 2004; Herzing et al., 2008; Xie et al., 2009). As discussed in the gas sensing section, the surface area and the porosity greatly influence the gas-solid interactions. Therefore, similar principles should be obeyed for the nanocatalysts design. The catalyst should have large surface to volume ratio so as to expose majority of active sites and facilitate the gas adsorption at the same time. Several nanocatalysts in the powder form have been discovered to have high efficiency toward CO oxidation. For example, Co_3_O_4_ nanocrystals have been demonstrated to achieve 100% catalytic CO conversion to CO_2_ at temperature as low as −77°C and the activity can be maintained in moisture stream of feed gas (Xie et al., 2009). Before Co_3_O_4_ nanopowders are demonstrated, the catalytic reaction of CO oxidation usually involves gold nanoparticles as the catalyst. Haruta et al. systematically studied gold nanoparticles supported on various metal oxides and their catalytic CO oxidation performance (Haruta et al., 1993). The supported gold nanoparticles are found to become the sites for the reversible adsorption of CO. At the same time, they are able to increase the amount of oxygen adsorbed on the support oxides. A generally accepted model for catalytic CO oxidation mechanism is that the adsorbed CO molecules on Au nanoparticles are going to travel toward the support oxides and react with adsorbed oxygen to form carbonate species. The decomposition of the carbonate intermediate gives rise to carbon dioxide as the final product. With the development of solution chemistry, many Co_3_O_4_ nanostructures with various geometries have been prepared (Hu et al., 2008b, 2010; Teng et al., 2011) and different geometric anisotropy is believed to influence the catalytic activity by different crystal facets exposed. Other materials systems such as CeO_2_(Ho et al., 2005; Pan et al., 2008; Wu et al., 2012), MnO_2_ (Liang et al., 2008; Wang et al., 2009; Ching et al., 2011), CuO (Jernigan and Somorjai, 1994; Feng and Zheng, 2010) have also been investigated. A nice review article by Royer et al. can be referred to for more details on transition metal oxide used for catalytic CO oxidation (Royer and Duprez, 2011).

As the simplest hydrocarbon, methane is widely used in power generation but it is also a greenhouse gas 20 times effective than carbon dioxide (Farrauto, 2012). The low temperature oxidation of methane is thus, critical for emission regulation and control (Choudhary et al., 1999; Eguchi and Arai, 2001). Cargnello et al. reported a hierarchical design of Pd@CeO_2_ core-shell catalyst which is deposited onto a modified hydrophobic Al_2_O_3_ (Cargnello et al., 2012). Metal-support interaction was discovered to cause complete conversion of methane below 400°C (Cargnello et al., 2012) and the Pd@CeO_2_ core-shell structure has excellent thermal stability (Cargnello et al., 2009; Adijanto et al., 2013). For nitrogen oxide removal, selective catalytic reduction (SCR) and NOx storage and reduction (NSR) are widely used technologies. In SCR, reducing gases such as ammonia (Wu et al., 2008; Metkar et al., 2011) or hydrocarbon (Amiridis et al., 1996; Shibata et al., 2003; Brosius and Martens, 2004; Liu et al., 2012a,b,c) are utilized to react with nitrogen oxide to produce nitrogen and water. However, NSR technique is slightly more complex (Takahashi et al., 1996; Liu and Gao, 2011). The NSR catalyst usually consists of storage materials such as barium oxide or carbonate, noble metal (Pt, Pd) and porous support (Al_2_O_3_). The nitric oxide (NO) will be first converted into NO_2_ through catalytic oxidation by Pt during lean condition where oxygen is rich. The barium component absorbs the nitrogen oxide and transforms into barium nitrate. When switched to rich cycle where fuel is sufficient, the decomposition of barium nitrate and reduction take place simultaneously to convert nitrogen oxide into nitrogen and water.

In practical application for catalytic gas treatment, the nanocatalysts must be deposited onto substrate to make a catalytic reactor or converter. Compared with powder form catalyst, catalytic reactors are generally more efficient and cost-effective due to several advantages enabled by the monolithic configuration such as low pressure drop, high surface area, efficient mass transfer and relatively low catalytic materials usage (Williams, 2001). However, the traditional procedures for constructing a catalytic reactor usually involve the wash coating of nanocatalysts (Irandoust and Andersson, 1988; Choi et al., 1996). The wash-coated nanopowders lack well-defined structural features and geometrical size, which leads to compromised materials usage and lower catalytic activity. Another issue associated with the catalytic reactor is the inevitable use of noble metals. Although some other nanomaterials have been discovered to exhibit excellent catalytic performance, their thermal and mechanical stability under practical working condition are usually far from satisfactory, which prevents them from large scale application in industry. A new nanomaterial manufacturing process is therefore, necessary to realize rational configuration of nanostructure without compromised catalytic activity.

**Figure 9:**
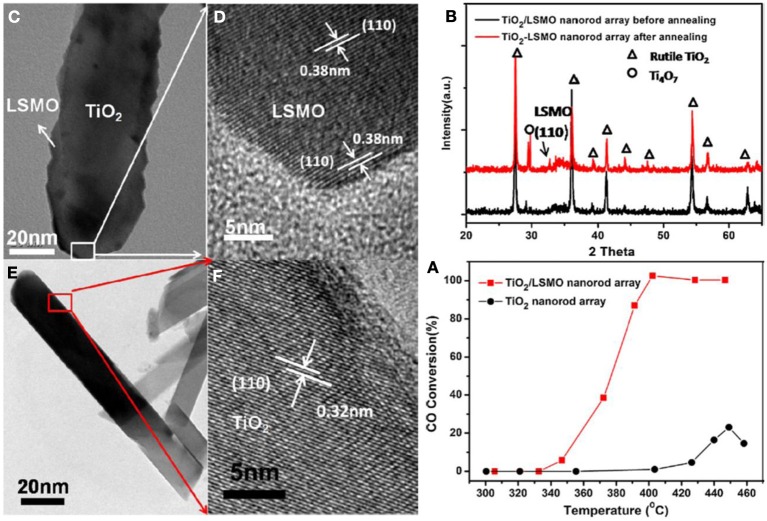
**Rutile TiO_2_/LSMO nanorod arrays for CO oxidation. (A)** Catalytic CO oxidation performance of two dimensional TiO2/LSMO composite nanowires array. **(B)** X-ray diffraction pattern of the nanowires array before and after thermal annealing. **(C,D)** TEM and HRTEM characterization of TiO_2_/LSMO composite nanowires. **(E,F)** TEM and HRTEM characterization of TiO2 nanowires. [Reprinted with permission from reference Guo et al. (2012). Copyright 2012 Elsevier].

Although many types of nanostructures have been proved to have great catalytic activity in their powder-form, devices based on hierarchical nanowire arrays have rarely been reported in the catalytic gas treatment. This is because of the lack of synthetic strategy to rationally integrate nanocatalysts on a large scale. We recently demonstrated the possibility to prepare a catalytic converter by hierarchical assembly of one-dimensional nanowires as building blocks. Two-dimensional planar assembly was first investigated and ordered TiO_2_/(La,Sr)MnO_3_ composite nanorod array catalysts have been successfully fabricated as shown in Figure 9 (Guo et al., 2012). Instead of using template, the TiO_2_ nanorod arrays on silicon or glass substrate were prepared by a one-pot facile hydrothermal synthesis. The radio-frequency (RF) magnetron sputtering method was applied to deposit a uniform coating of (La,Sr)MnO_3_ on the TiO_2_ nanorod surface. TiO_2_ is a traditional oxide support for immobilizing noble metal catalyst, while it can be served as a catalyst itself in photo-exited reactions due to the semiconducting nature. In our experiments the hydrothermally prepared TiO_2_ nanorod arrays were found to be rutile TiO_2_ in crystal phase. But the as-prepared TiO_2_/LSMO composite nanorod array does not exhibit perovskite LSMO peak due to the poorly crystallized as-deposited LSMO shell. The thermal annealing at 800°C improved the crystallinity and X-ray diffraction results revealed that {110} planes are the dominant crystal facet in the LSMO shell. The ratio between La and Sr in LSMO is close to 1:1. An important rationale behind such materials combination is that perovskite have the oxidation activity comparable to that of the platinum group metal catalysts. It has been demonstrated to have an improved performance for automobile exhaust emission control such as lean NO_*X*_ trapping (Kim et al., 2010) CO oxidation was used as a probe reaction to characterize the catalytic performance of the hierarchical TiO_2_/LSMO composite nanorod arrays. The maximum CO conversion efficiency of pure rutile TiO_2_ nanorod array was very low and was only around 20%, while the TiO_2_/LSMO composite nanorod array achieved 100% CO conversion efficiency at 400°C. The catalytic performance was also better than that of perovskite LSMO thin film catalyst from literature (Karita et al., 2007).

**Figure 10:**
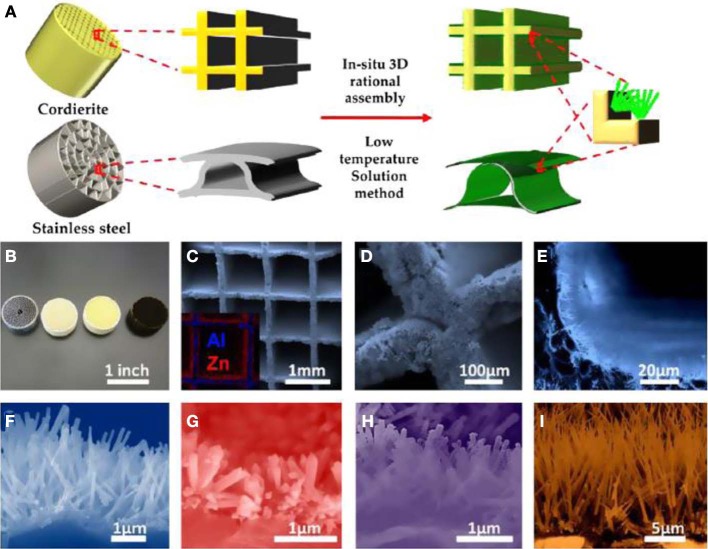
**Three-dimensional integration of various nanowires onto honeycomb substrates for a prototype high efficient catalytic converter. (A)** Schematic illustration of in-situ three dimensional assembly of nanowires onto monolithic honeycomb substrates to develop a prototype catalytic converter. **(B)** Photographs of honeycomb substrates before and after nanostructure coverage. **(C–E)** SEM characterization of nanowires array-based catalytic converter at different magnification. Inset of **(C)** EDS mapping of ZnO nanowires based honeycombs. **(F–I)** SEM cross-section views of ZnO nanowires(F), TiO_2_ nanorods(G), CeO_2_ nanotubes(H) and Co3O_4_ nanowires(I). [Reprinted with permission from reference Guo et al. (2013). Copyright 2013 Elsevier].

With successful demonstration of two-dimensional assembly, we further explored the possibility of three-dimensional integration of nanowires onto the ceramic cordierite honeycomb substrate to develop a prototype catalytic converter (Guo et al., 2013). Figure 10 illustrates the materials synthesis process and the electron microscopy characterization of the nanostructures. The three-dimensional ordered nanoarchitectures provide well-defined geometry with controlled porosity and surface area which allows for efficient gas diffusion and facilitates the gas-solid interaction. Different metal oxide nanowire arrays with various morphologies including ZnO nanowires, TiO_2_ nanorods, Co_3_O_4_ porous nanowires and CeO_2_ nanotubes have been *in situ* grown on the honeycomb substrate by a facile hydrothermal synthesis. The nanowires array based catalytic honeycombs were discovered to have good thermal stability under 800°C for 100 h and decent mechanical robustness exposed to constant air flow with rate of 50 L/min for 10 days. Pt nanoparticles were then wash coated onto the nanostructures with controlled weight ratio (Pt/nanowires = 1 wt %). The catalytic performance of these nanowire arrays based honeycombs are studied by using CO oxidation as the probe reaction. Comparable and even better catalytic performances were achieved compared to traditional powder catalyst prepared by washcoating. However, the materials usages of both support metal oxide and the noble metal were reduced by 10–40 folds with sacrificing the catalytic performance. It is therefore, plausible to conclude that the ordered nanoscale geometry with well-defined shape and arrangement will reduce the manufacturing cost of catalyst preparation by improving the materials utilization efficiency. The low materials consumption as well as the maintained high catalytic activity makes the hierarchical nanostructure based honeycombs a potentially promising design for better catalytic converter. Another advantageous feature of the nanostructured catalytic honeycombs is that it allows for more control over the catalytic performance by tailoring nanoscale structures. The well-defined geometry and special arrangement help elucidate the structure-property relation, which in this case is the correlation between nanostructure and the catalytic activity, and thus, provide rationales for catalytic materials design. For instance, in our nanostructured honeycombs, we are able to tune the catalytic CO oxidation performance by adjusting different shape and structure of the ZnO support. Specifically, longer ZnO nanowires allow for more uniform dispersion of Pt nanoparticles that favors the reaction kinetics. Besides, when the aspect ratio of single crystalline ZnO nanowire decreases and the shape transforms from one-dimensional nanowires to two-dimensional nanoplates, the catalytic activity toward CO oxidation is much improved. This can be ascribed to the different interaction of Pt nanoparticles with specific crystal facets of ZnO nanostructures. The interaction of Pt with ZnO (0001) is found to favor the catalytic CO oxidation (Roberts and Gorte, 1990; Petrie and Vohs, 1994). When the ZnO (0001) planes are highly involved in the nanostructure, the catalytic performance will be improved.

In this section, we start with typical catalyst materials toward different polluting gas and introduced the progress in nanocatalysts preparation in different gas treatments. Later we summarized the recent research effort on integration of nanoscale building blocks to achieve hierarchical nanostructures for catalytic device fabrication and illustrate the advantages of ordered nanostructure arrangement over the traditional wash-coated powder catalyst. For the future development of hierarchical nanostructured catalytic converter, however, the design of hierarchical nanostructures with rational combination of multiple components targeted at complex chemical reactions is still challenging. Much research effort should be devoted regarding better materials selection, further reduction of noble metal usage and industrial scale fabrication.

## Concluding remarks

Hierarchical nanostructures have been extensively studied worldwide owing to the potential applications in many research and industrial areas. The review mainly summarizes the latest progress in hierarchical nanostructures developed for environmental applications particularly on water treatment, biosensing and environmental gas sensing, monitoring and catalytic conversion. In each section, we elucidate the physics behind the working mechanism and discuss the related hierarchical nanostructures design from simple random free standing nanostructures toward ordered arrangement of nanoscale building blocks with increased complexity in both geometry and chemical components. The advantages from hierarchical assembly have been discussed in each specific application.

In photocatalytic water treatment, we focused on the synthetic strategies developed so far to prepare hierarchical nanostructures and the rationales behind the materials combination based on the relevant physics in the water treatment process. Specifically, we reviewed the preparation of hollow and urchin-like architecture with either single or multiple compositions, which greatly improve the surface area and favors the reaction kinetics. Band gap engineering and plasmonic photocatalysis were also discussed for high efficient catalytic activity especially under visible light illumination. In environmental gas sensing and monitoring, we started with the fundamentals of gas sensing mechanisms followed by hierarchical materials design to achieve high sensitivity and selectivity. Then we further discussed the device fabrication toward gas sensing application in different scenarios ranging from toxic gas detection, environmental gas monitoring to gas sensor applied in harsh environments. Lastly, we summarized the latest research progress in hierarchically nanostructured catalytic device fabrication which is important to catalysis industry owing to better materials utilization efficiency, particularly the reduced usage of noble metal, and the possibility of adjusting catalytic activity by tailored nanoscale architectures.

Finally, driven by the novel properties that nanostructures possess and the emergent needs of developing low-cost and highly efficient devices for environmental applications, hierarchically nanostructured materials and related devices have become a promising class of candidates for the next generation technology toward environmental sustainability. It is worth pointing out, however, the nanostructure geometric arrangement and multifunctional coupling present two of the most obvious important challenges from hierarchical structuring perspective, which if solved will enable a great deal of cost-effective, energy efficient, and multifunctional practical devices and solutions for environmental protection and control. Toward addressing these structuring and functioning challenges, it should be stressed that enhanced fundamental understanding and rationally deposited new principles or theories are in great need on the (dis-)similar nanostructure assembly, growth, and processing. On the other hand, the functional coupling and synergy between (dis-)similar nanostructures are worth tackling as the pre-requisite understanding toward multifunctional nanostructure devices and systems suitable for various practical applications. Another concurrent need for hierarchical nanostructuring is the validation and improvement upon the green, scalable, and sustainable nanomaterials manufacturing, which is becoming a more and more important subject with the further development of nanotechnology toward realistic technology platforms with added values, novel functions, and effective operations.

### Conflict of interest statement

The authors declare that the research was conducted in the absence of any commercial or financial relationships that could be construed as a potential conflict of interest.
